# Comparison of efficacy and prognosis between neoadjuvant chemotherapy and adjuvant chemotherapy in penile cancer patients with regional lymph node metastasis

**DOI:** 10.3389/fonc.2025.1611077

**Published:** 2025-12-08

**Authors:** Ruijian You, Yongpeng Ji, Xiao Yang, Qiang Zhao, Yudong Cao, Jinchao Ma, Ziyi Yu, Yushuang Cui, Jinduo Ma, Ruojing Li, Liuyang Miao, Yong Yang, Peng Du, Shuo Wang

**Affiliations:** Key Laboratory of Carcinogenesis and Translational Research (Ministry of Education), Urological Department, Peking University Cancer Hospital & Institute, Beijing, China

**Keywords:** penile squamous cell carcinoma, lymph node metastasis, neoadjuvant chemotherapy, adjuvant chemotherapy, progression-free survival, overall survival

## Abstract

**Introduction:**

The comparative efficacy of neoadjuvant chemotherapy (NAC) and adjuvant chemotherapy (AC) in patients with lymph node metastatic penile squamous cell carcinoma (PSCC) remains unclear. This study aims to evaluate the differences in efficacy and survival outcomes between NAC and AC in PSCC patients with lymph node metastasis.

**Methods:**

We conducted a retrospective analysis of penile cancer patients treated at Peking University Cancer Hospital between May 2015 and December 2023. All patients had lymph node metastasis and underwent lymph node dissection. 11 patients received NAC, while 14 received AC. Kaplan-Meier survival curves and multivariate Cox regression models were used to analyze baseline characteristics, progression-free survival (PFS), overall survival (OS), and associated risk factors.

**Results:**

There was no statistical difference in the baseline characteristics of patients between the two groups. In the NAC group, 4 patients achieved partial response (PR), 1 achieved complete response (CR), 4 achieved progression disease (PD), and 2 had stable disease (SD). After a median follow-up of 65.1 months, the NAC group had a shorter PFS compared to the AC group (*P* = 0.029), while no significant difference was observed in OS (*P* = 0.268). Multivariate Cox regression analysis identified the presence of cM stage as an independent predictor of both PFS and OS.

**Conclusion:**

Penile cancer with lymph node metastasis typically carries a dismal prognosis. NAC substantially diminishes tumor size and facilitates surgical excision, whereas AC is more effective in controlling postoperative tumor progression. However, there was no significant difference in overall survival between the two treatment strategies.

## 1 Introduction

Penile squamous cell carcinoma (PSCC) is a rare urogenital malignancy associated with poor prognosis in advanced stages (1). The high-risk factors for penile cancer include obesity, poor hygiene and socioeconomic status, phimosis, a higher number of sexual partners, HPV infection, and smoking (2). Although PSCC accounts for less than 1% of all malignancies in Western countries, its incidence is increasing in developing regions (3). Localized invasive penile cancer represents approximately 40% of cases, with a 5-year survival rate of 90%. However, prognosis worsens significantly once metastasis occurs (4). Patients with unilateral inguinal lymph node metastasis involving ≤2 nodes have a 5-year OS of approximately 80%, whereas those with bilateral or pelvic lymph node involvement exhibit a markedly lower a 5-year OS of 10-20% (5). The European Association of Urology (EAU) and the National Comprehensive Cancer Network (NCCN) guidelines advocate for organ-preserving surgery in cases of intraepithelial neoplasia and localized invasive penile cancer. However, this approach is not suitable for patients with inguinal lymph node metastasis (6, 7). The prognosis of advanced PSCC is influenced by multiple factors, including histopathological subtype, neurovascular invasion, and extracapsular lymph node extension. In such cases, systemic therapy is essential, with chemotherapy being the most commonly utilized treatment modality (8, 9). Other systemic treatment options include immunotherapy and cytokine therapy, though chemotherapy remains the mainstay.

In the context of neoadjuvant chemotherapy (NAC), a multicenter retrospective study evaluating platinum-based NAC in PSCC patients with lymph node metastasis reported a median OS of 37.0 months, a median progression-free survival (PFS) of 26.0 months, and an objective response rate (ORR) of 57.2%. Patients who responded to NAC demonstrated prolonged OS, although 17% experienced chemotherapy-related adverse events (9). A systematic review suggests that NAC achieves an ORR of approximately 50%. Compared to non-paclitaxel platinum-based regimens, paclitaxel-based chemotherapy is associated with a higher pathological complete response (pCR) rate but also increased chemotherapy-related toxicity (10). Furthermore, studies indicate that patients achieving an objective response to NAC exhibit improved survival outcomes (11, 12). In terms of AC, research has demonstrated its efficacy in pN3 patients, with a median OS improvement of 11.6 months compared to those who did not receive AC (13–17). A recent meta-analysis demonstrated that the 5-year survival rate for patient receiving NAC with cN1–3 disease is 29%, with a pathological complete response (pCR) rate of 13% and an objective response rate of 51%. However, for patients with N1–2 disease, there is insufficient evidence in improving survival. In inguinal cN3 disease not immediately surgically resectable, NAC may induce a response rate to allow subsequent inguinal lymph node dissection. Survival analysis of patients receiving adjuvant chemotherapy indicates a 12-month survival rate of 85%.Both NAC and AC contribute to improved prognosis and survival in PSCC with lymph node metastasis, yet few studies have directly compared their efficacy (12). Some meta-analyses suggest that AC may offer superior OS and PFS compared to NAC, with a similar adverse event profile (11, 13, 18, 19). Given the paucity of comparative studies, this research aims to evaluate the differences in efficacy between NAC and AC.

## 2 Materials and methods

### 2.1 Study population

We included 25 patients with penile squamous cell carcinoma (PSCC) who underwent neoadjuvant chemotherapy (NAC) or adjuvant chemotherapy (AC) at Peking University Cancer Hospital between May 2015 and December 2023. Among them, 11 patients received NAC, while 14 received AC. Clinical and pathological staging was performed according to the 8th edition of the American Joint Committee on Cancer (AJCC) staging system (20). Histopathological grading followed the Broders classification, which categorizes PSCC into well-differentiated, moderately differentiated, and poorly differentiated subtypes (21). Eligible patients were aged 34–66 years, had histologically confirmed PSCC, and were clinically staged as TxN+Mx. All patients had inguinal or pelvic lymph node metastasis confirmed by physical examination, computed tomography (CT), or positron emission tomography-computed tomography (PET-CT). Patients who have received radiotherapy are excluded. This study adhered to the principles outlined in the 2013 revision of the Declaration of Helsinki, and written informed consent was obtained from all participants. This study did not perform a formal sample size calculation prior to data collection. Given the retrospective nature of the analysis and the availability of patients with lymph node metastasis who met the inclusion criteria, a convenience sample was used. The small sample size is acknowledged as a limitation of the study, which may impact the statistical power of the findings.

### 2.2 Treatment protocol

The systemic treatment regimen consisted of paclitaxel, ifosfamide and cisplatin (TIP). Paclitaxel (175 mg/m²) was administered on day 1, while ifosfamide (1200 mg/m²) and cisplatin (25 mg/m²) were given on days 1-3. The regimen was repeated every 3 weeks. Tumor response was evaluated every two cycles using pelvic contrast-enhanced computed tomography (CT) and assessed according to the Response Evaluation Criteria in Solid Tumors (RECIST) version (22). Objective response to NAC was defined as a reduction or disappearance of metastatic lymph nodes or increased mobility of lymph nodes on physical examination or CT. Non-response was defined as stable or progressive disease. Consolidative lymph node dissection was performed 3–4 weeks after NAC, while AC was initiated three weeks postoperatively.

### 2.3 Objectives

The primary endpoints were progression-free survival (PFS) and overall survival (OS). PFS was defined as the time from surgery to disease progression or death from any cause, with patients who were alive and progression-free at the last follow-up censored. OS was defined as the time from diagnosis to death from any cause.

### 2.4 Statistical analysis

Data were analyzed using SPSS version 27.0. Descriptive statistics were reported as mean ± standard deviation (SD), median (range), or frequency (percentage). The number of positive lymph nodes between the NAC and AC groups was compared using the non-parametric Mann-Whitney U test. Kaplan-Meier survival curves were generated, and between-group comparisons were conducted using the log-rank test. Multivariate Cox regression analysis was performed to identify risk factors associated with survival. A *P*-value <0.05 was considered statistically significant. The statistical power of the study was calculated using G*Power software.

## 3 Results

### 3.1 Patient characteristics

This study included 25 histologically confirmed PSCC patients at Peking University Cancer Hospital between May 2015 and December 2023, all of whom received TIP chemotherapy. The NAC group consisted of 11 patients, while the AC group comprised 14 patients, with a mean age of 51.4 years. No significant differences were observed between the two groups in terms of age, BMI, smoking history, chronic disease comorbidities, alcohol consumption, local treatment, histopathological grade, clinical N stage, clinical M stage, or the number of chemotherapy cycles (*P* > 0.05, **Table 1**). There was no statistically significant difference in the number of postoperative positive lymph nodes between the two groups. The median number of positive nodes was 1.0 (IQR, 1.0-3.5) in the NAC group and 1.5 (IQR, 1.0-4.5) in the AC group (*P* = 0.720).

**Table 1:** Baseline characteristics of patients in the NAC and AC groups.

Characteristics	ALL	NAC	AC	*P*
Patients (n)	25 (100%)	11 (44.0%)	14 (56.0%)	–
Age (years)	51.4±10.7	51.4±12.3	51.4±9.8	0.988
BMI (kg/m2)	27.1±3.8	25.8±3.7	28.1±3.7	0.134
History of smoking				0.434
Current/Prior	9 (36.0%)	6 (54.5%)	10 (71.4%)	
Never	16 (64.0%)	5 (45.5%)	4 (28.6%)	
History of alcoholism				0.407
Current/Prior	18 (72.0%)	2 (18.2%)	5 (35.7%)	
Never	7 (28.0%)	9 (81.8%)	9 (64.3%)	
Chronic disease comorbidities				0.435
YES	14 (56.0%)	6 (54.5%)	5 (35.7%)	
NO	11 (44.0%)	5 (45.5%)	9 (64.3%)	
Treatment of Penile Tumors				0.431
Partial penectomy	15 (60.0%)	5 (45.5%)	10 (71.4%)	
Radical penectomy	3 (12.0%)	2 (18.2%)	1 (7.1%)	
Local excision	7 (28.0%)	4 (16.4%)	3 (21.4%)	
Broders’classifcation				0.465
Well differentiated	6 (24.0%)	3 (27.3%)	4 (28.6%)	
Moderately differentiated	14 (56.0%)	5 (45.5%)	9 (64.3%)	
Poorly differentiated	4 (16.0%)	3 (27.3%)	1 (7.1%)	
cN stage				0.775
N1	6 (24.0%)	3 (27.3%)	3 (24.0%)	
N2	11 (44.0%)	4 (36.4%)	7 (50.0%)	
N3	8 (32.0%)	4 (36.4%)	4 (28.6%)	
cM stage				0.775
M0	23 (92%)	9 (81.8%)	14 (100%)	
M1	2 (8.0%)	2 (18.2)	0 (0%)	
Cycles of chemotherapy				0.744
2	1 (4.0%)	1 (9.1%)	0 (0%)	
3	8 (32.0%)	4 (36.4%)	4 (28.6%)	
4	14 (56.0%)	5 (45.5%)	9 (64.3%)	
6	2 (8.0%)	1 (9.1%)	1 (7.1%)	

Data are shown as mean ± SD or n (%). P values were calculated using t test for continuous variables and χ^2^ or Fisher’s exact test for categorical variables. NAC, neoadjuvant chemotherapy; AC, adjuvant chemotherapy; BMI, body mass index.

### 3.2 Follow-up

In the NAC group, the clinical response was evaluated before surgery. A complete response (CR) was observed in 1 patient (9.1%), and a partial response (PR) was observed in 4 patients (36.4%). Five patients (45.4%) had stable disease (SD), and one patient (9.1%) had progressive disease (PD).

Therefore, the overall response rate (ORR), defined as the sum of CR and PR, was 45.5%.

Five patients completed four cycles of TIP, while one received six cycles due to persistent unresectable disease after four cycles. Two patients progressed after two cycles, and four received only three cycles due to chemotherapy-related toxicity. All patients in the NAC group underwent lymph node dissection: six patients underwent unilateral inguinal lymph node dissection (ILND), two underwent bilateral ILND, two underwent unilateral ILND with pelvic lymph node dissection (PLND), and one underwent bilateral ILND with PLND.

In the AC group, five patients underwent unilateral ILND, three underwent bilateral ILND, four underwent unilateral ILND with PLND, and two underwent bilateral ILND with PLND. Nine patients completed four cycles of chemotherapy, one completed six cycles, and three completed only three cycles due to toxicity.

### 3.3 Survival analysis

The median follow-up time was 65.1 months. In the NAC group, 8 patients achieved recurrence or metastasis: 2 patients had recurrence in the inguinal lymph nodes, 1 patient had recurrence in both inguinal and pelvic lymph nodes, 2 patients had lung metastases, and 2 patients had bone metastases. Six patients died, all related to penile cancer. Among those who received adjuvant chemotherapy, 3 experienced recurrence or metastasis: 1 case of local inguinal recurrence, 1 case of contralateral inguinal lymph node metastasis, and 1 case of pelvic lymph node metastasis with bone metastasis. Four patients died, including three due to penile cancer-related causes and one from a non-penile cancer-related cause.

**Figure 1:**
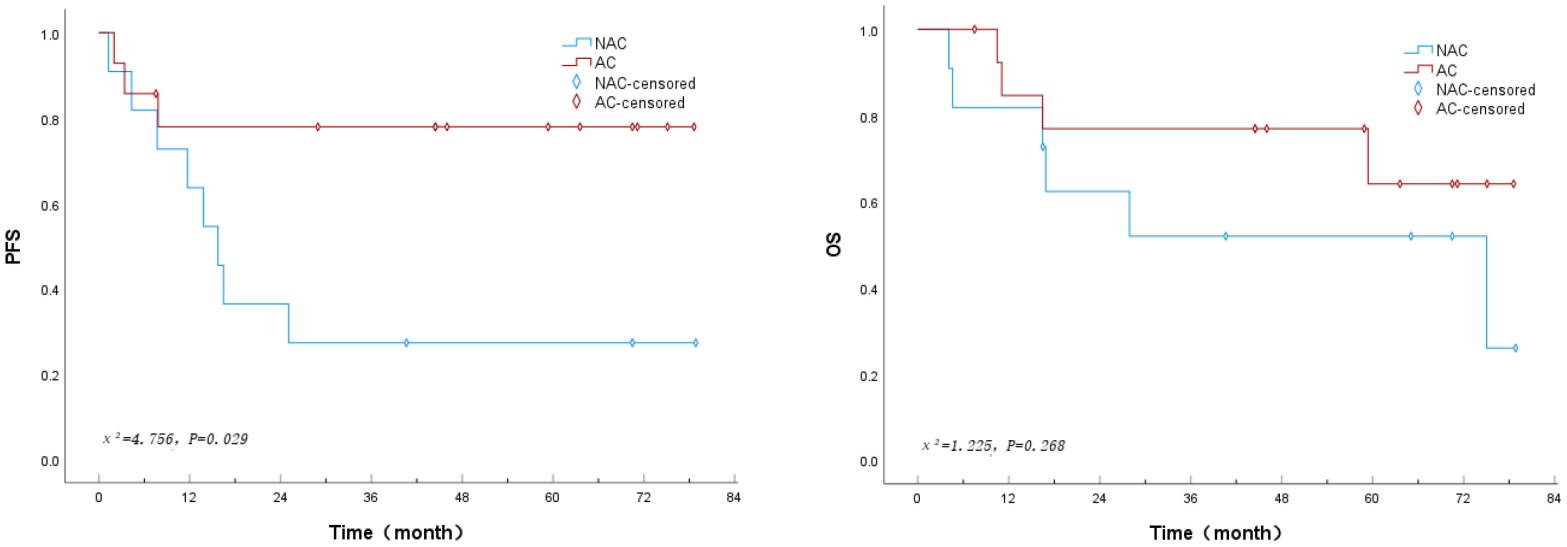
Kaplan–Meier curves of progression-free survival (PFS) and overall survival (OS) in patients receiving neoadjuvant chemotherapy (NAC) versus adjuvant chemotherapy (AC). **(A)** Progression-free survival (PFS): patients in the AC group showed significantly longer PFS than those in the NAC group (HR 3.963, 95% CI 1.044–15.045; P = 0.029). **(B)** Overall survival (OS): no statistically significant difference was observed between the two groups (HR 2.010, 95% CI 0.566–7.141; P = 0.268). Censored cases are indicated by marks on the curves. NAC, neoadjuvant chemotherapy; AC, adjuvant chemotherapy; PFS, progression-free survival; OS, overall survival.

The Kaplan-Meier method was used to evaluate survival in both groups. The results showed that the median progression-free survival (PFS) in the neoadjuvant chemotherapy group was 15.7 months (95% CI: 10.6–20.9), while the median PFS in the adjuvant chemotherapy group was not reached, and the overall median PFS was also not reached. The median overall survival (OS) in the neoadjuvant chemotherapy group was 75.0 months (95% CI: 12.4–137.6), whereas the median OS in the adjuvant chemotherapy group was not reached, with an overall median OS of 75.0 months (95% CI: NR–NR). The PFS in the neoadjuvant chemotherapy group was significantly inferior to that in the adjuvant chemotherapy group (HR=3.963, 95% CI 1.044-15.045; *P* = 0.029], while there was no significant difference in OS between the two groups (HR=2.010, 95% CI 0.566-7.141; *P* = 0.268) (**Figure 1**). The HR for PFS in the NAC group was 3.963 (95% CI 1.044-15.045, *P* = 0.029), suggesting a significant benefit for AC. With a statistical power of 75.74%, this result suggests a moderate likelihood of detecting the observed effect. However, the HR for OS was 2.010 (95% CI 0.566-7.141, *P* = 0.268), which did not reach statistical significance. The lower power for OS (25.39%) indicates that the study may not have had sufficient capacity to detect smaller, clinically relevant differences in OS between the two treatment groups.

Given that factors such as age at diagnosis, BMI, smoking history, comorbidities, alcohol consumption, clinical N stage, type of penile surgery, histological grade, clinical stage, number of chemotherapy cycles, and receipt of neoadjuvant chemotherapy may significantly impact patient prognosis, a Cox proportional hazards model was applied for regression analysis. For PFS, univariate analysis identified neoadjuvant chemotherapy and the presence of chronic diseases as significant factors. Multivariate analysis further demonstrated that age, presence of chronic diseases, clinical M stage, and the number of chemotherapy cycles were independent prognostic factors for PFS (**Table 2**). Regarding OS, univariate analysis indicated an association between the presence of chronic diseases and OS. Multivariate analysis incorporating relevant variables confirmed that age, presence of chronic diseases, and clinical M stage were independent prognostic factors for OS (**Table 3**).

**Table 2:** Univariate and multivariate cox regression analysis of PFS in patients receiving neoadjuvant chemotherapy (NAC) and adjuvant chemotherapy (AC).

Influencing factors	Univariate analysis			Multivariate analysis		
	P-value	HR	95% CI	P-value	HR	95%CI
NAC vs. AC	0.043	0.252	0.066~0.958			
Age	0.868	1.005	0.949~1.064	0.026	0.902	0.824~0.988
Smoking History	0.389	1.795	0.474~6.792			
Alcohol History	0.397	1.705	0.496~5.864			
Chronic Disease	0.017	5.175	1.347~19.879	0.003	85.730	4.729~1554.241
BMI	0.168	0.889	0.751~1.051			
Treatment of Penile Tumors	0.444	1.620	0.471~5.573			
Broders’classifcation	0.681	1.199	0.505~2.846			
cN stage	0.339	0.677	0.305~1.505			
cM stage	0.096	3.690	0.793~17.170	0.003	175.236	5.982~5133.269
Cycles of chemotherapy	0.066	0.496	0.235~1.048	0.004	0.327	0.154~0.696

Cox proportional hazards regression analysis was performed to evaluate the impact of clinical and demographic factors on progression-free survival (PFS) in patients treated with NAC or AC. Univariate analysis assessed each variable individually, while multivariate analysis adjusted for significant covariates. Hazard ratios (HR) and 95% confidence intervals (CI) are reported. Variables with P-values <0.05 in multivariate analysis were considered independent predictors of PFS. Notably, age, chronic disease status, cM stage, and number of chemotherapy cycles were significantly associated with PFS. NAC, neoadjuvant chemotherapy; AC, adjuvant chemotherapy; BMI, body mass index; HR, hazard ratio; CI, confidence interval; PFS, progression-free survival.

**Table 3:** Univariate and multivariate cox regression analysis of OS in neoadjuvant and adjuvant chemotherapy.

Influencing factors	Univariate analysis			Multivariate analysis		
	P-value	HR	95% CI	P-value	HR	95%CI
NAC vs. AC	0.281	0.498	0.140~1.768			
Age	0.860	0.995	0.938~1.055	0.078	0.936	0.870~1.008
Smoking History	0.235	2.568	0.542~12.172			
Alcohol History	0.137	2.567	0.741~8.890			
Chronic Disease	0.012	7.530	1.562~36.295	0.007	12.511	2.015~77.683
BMI	0.244	0.898	0.750~1.067			
Treatment of Penile Tumors	0.108	1.559	0.814~2.985			
Broders’classifcation	0.770	0.874	0.353~2.159			
cN stage	0.120	0.512	0.220~1.190			
cM stage	0.520	1.984	0.246-16.028	0.020	0.341	0.137~0.846
Cycles of chemotherapy	0.208	0.566	0.234~1.372			

Hazard ratios (HRs) and 95% CIs for overall survival are shown for clinical and pathological factors in patients treated with neoadjuvant chemotherapy (NAC) or adjuvant chemotherapy (AC). HRs were estimated using Cox proportional hazards regression. Variables significant in univariate analysis (p<0.05) were entered into the multivariate model. In multivariate analysis, the presence of chronic disease and cM stage remained independent prognostic factors for overall survival. NAC, neoadjuvant chemotherapy; AC, adjuvant chemotherapy; BMI, body mass index; HR, hazard ratio; CI, confidence interval; PFS, progression-free survival.

## 4 Discussion

Although penile cancer has a relatively low incidence in developed countries, it remains a significant concern in developing regions. In this study, the median age at initial diagnosis was 51 years, which is considerably younger than the reported median age of 70 years in developed countries (23). This suggests a notable difference in disease onset between populations. Penile cancer most commonly presents as a painless mass or ulceration. Garg et al. reported that 71% of patients presented with lymph node enlargement at the time of consultation (24, 25). Similarly, Chalya et al. found that 68.6% of patients were diagnosed at an advanced stage, with 56% classified as stage III, 12.7% as stage IV, and 65% having lymph node metastases (26). In our study, all included patients were lymph node-positive, with more than 70% classified as N2–N3, indicating that most patients were diagnosed at a late stage. This may be related to the limited awareness of penile squamous cell carcinoma (PSCC) among patients in China, as well as reluctance to seek medical attention due to social stigma. Both the NCCN and EAU guidelines recommend neoadjuvant chemotherapy as the first-line treatment for patients with enlarged or matted inguinal lymph nodes (27, 28). In a clinical trial conducted by Pagliaro et al., the neoadjuvant chemotherapy regimen consisted of four cycles of the TIP regimen, with an objective response rate (ORR) of 50% and a pathological complete response (pCR) rate of 10% (29). Additionally, a study by Mailankody et al. included 68 patients with advanced cancer, among whom 13 received neoadjuvant chemotherapy, achieving a response rate of 54% (23). A meta-analysis by Yi et al. reported that the ORR for taxane-plus-platinum combination therapy was approximately 57%, while non-taxane-plus-platinum regimens had an ORR of 54% (10). In our study, the ORR in the neoadjuvant chemotherapy group was 45.5%, which is lower than that reported in Pagliaro et al.’s study. Notably, 72.8% of patients in our neoadjuvant chemotherapy group were classified as cN2–N3, a higher proportion than in Pagliaro et al.’s study, which may be a key factor contributing to the lower ORR observed in our cohort. Additionally, Pagliaro et al.’s study primarily included Western populations and did not enroll Asian patients, whereas our study exclusively involved Chinese patients. Currently, no studies have reported whether ethnicity affects treatment outcomes. Therefore, we cannot conclude that Chinese PSCC patients have a poorer prognosis than Western patients when matched for clinical stage and treatment regimen.

A key concern in comparing neoadjuvant and adjuvant therapies is the potential for baseline differences in tumor burden between the groups. In our study, we specifically analyzed the postoperative pathological lymph node status, a powerful indicator of disease burden. Our finding that there was no significant difference in the number of positive nodes between the NAC and AC groups suggests that the observed survival differences are less likely attributable to a confounding effect from the extent of nodal metastasis. Chemotherapy is an essential component of systemic therapy for lymph node-positive PSCC patients (6, 12, 27). Although several retrospective studies and meta-analyses have reported the role of chemotherapy in neoadjuvant and adjuvant settings (14, 30–34), and various guidelines recommend chemotherapy as a potential option for neoadjuvant or adjuvant therapy, there is currently a lack of large prospective clinical studies or meta-analyses demonstrating that neoadjuvant chemotherapy or adjuvant chemotherapy provides greater benefits in terms of progression-free survival or overall survival. This may be due to the relatively low incidence of penile cancer, making PSCC patients rare. However, a recent retrospective study that included 91 PSCC patients receiving systemic therapy highlighted a significant difference in overall survival (OS) between patients who received neoadjuvant chemotherapy and those who received adjuvant chemotherapy (P < 0.0001). The median OS for the neoadjuvant chemotherapy group was 14.4 months, while the median OS for the adjuvant chemotherapy group was 89.9 months (35). Previous studies, such as those by Nicolai et al., have also suggested that patients who receive adjuvant chemotherapy tend to experience later recurrences compared to those who receive neoadjuvant chemotherapy (13). Another meta-analysis, which included 1,197 patients, found no statistically significant differences in overall survival (OS) and progression-free survival (PFS) between patients receiving neoadjuvant and adjuvant chemotherapy. However, the conclusion leaned toward adjuvant chemotherapy providing better survival benefits compared to neoadjuvant chemotherapy (11),which is consistent with our findings. In our study, the median PFS for the neoadjuvant chemotherapy group was 15.7 months, while the median PFS for the adjuvant chemotherapy group was not reached. The median OS for the neoadjuvant chemotherapy group was 75.0 months, and the median OS for the adjuvant chemotherapy group was also not reached. The PFS in the neoadjuvant chemotherapy group (HR = 3.963, 95% CI 1.044-15.045; *P* = 0.029) was inferior to that in the adjuvant chemotherapy group, indicating that the risk of recurrence in the NAC group is approximately four times higher than that in the AC group, while there was no significant difference in OS between the two groups (HR = 2.010, 95% CI 0.566-7.141; *P* = 0.268). These results suggest that, compared to neoadjuvant chemotherapy, adjuvant chemotherapy offers benefits in PFS and shows a trend toward improved OS. Due to the relatively small sample size, the statistical power to detect significant differences in survival outcomes between the NAC and AC groups is limited. The observed hazard ratios (HRs) for progression-free survival (PFS) in this study, while clinically relevant, may not fully reflect the true effect sizes in a larger cohort. Specifically, the HR for PFS in the NAC group was 3.963 (95% CI 1.044-15.045, P = 0.029), indicating a potential benefit for AC, but the small sample size limits the power to detect more subtle differences in survival outcomes. The wide 95% confidence interval for the HR (1.044-15.045) indicates a broad range of possible effect sizes, which underscores the need for larger studies to refine the estimated effect of NAC versus AC on PFS and ensure more precise clinical decision-making.

Penile cancer has a relatively low overall incidence, and the proportion of patients receiving neoadjuvant chemotherapy and surgery is also relatively low. Therefore, the sample size in this study is small, which limits our ability to perform stratified analysis based on different lymphatic involvement. Additionally, the retrospective nature of the study may introduce biases. While the study observed a significant difference in progression-free survival (PFS) between the NAC and AC groups (HR = 3.963, P = 0.029), the study’s statistical power for OS was only 25.39%, which is low. This limited power suggests that the non-significant result for OS (HR = 2.010, P = 0.268) may be due to the insufficient ability to detect subtle differences in survival outcomes. Future studies with larger sample sizes are needed to provide more robust estimates of treatment effects and ensure adequate statistical power, particularly for OS. Furthermore, due to the lack of detailed documentation of adverse reactions in our data, we are unable to report on the adverse effects experienced by patients during neoadjuvant chemotherapy and adjuvant chemotherapy. Lymph node involvement before chemotherapy was determined by imaging rather than biopsy, which may influence the pathological complete response rate after systemic chemotherapy. Although we assessed progression after neoadjuvant chemotherapy using the RECIST criteria, all patients in our study underwent lymph node dissection after neoadjuvant chemotherapy. This means that patients who were unable to undergo surgical consolidation treatment after neoadjuvant chemotherapy were excluded from our study. Thus, our conclusions are more applicable to patients who can undergo consolidation lymph node dissection after neoadjuvant therapy. In future studies, we aim to expand the sample size, improve data collection, and obtain more comprehensive information for more detailed analysis.

## 5 Conclusion

In conclusion, while our study provides preliminary evidence of the potential benefits of adjuvant chemotherapy (AC) over neoadjuvant chemotherapy (NAC) in patients with penile squamous cell carcinoma with lymph node metastasis, the small sample size and low statistical power for OS limit the generalizability and confidence in our findings. Larger, prospective studies with formal sample size calculations and higher statistical power are needed to validate these results and refine treatment strategies.

## Data Availability

The raw data supporting the conclusions of this article will be made available by the authors, without undue reservation.
